# Muscle hypertrophy induced by myostatin inhibition accelerates degeneration in dysferlinopathy

**DOI:** 10.1093/hmg/ddv288

**Published:** 2015-07-23

**Authors:** Yun-Sil Lee, Adam Lehar, Suzanne Sebald, Min Liu, Kayleigh A. Swaggart, C. Conover Talbot, Peter Pytel, Elisabeth R. Barton, Elizabeth M. McNally, Se-Jin Lee

**Affiliations:** 1 Department of Molecular Biology and Genetics and; 2 Institute for Basic Biomedical Sciences, Johns Hopkins University School of Medicine, 725 North Wolfe Street, PCTB 803, Baltimore, MD 21205, USA,; 3 Department of Physiology, Perelman School of Medicine and; 4 Department of Anatomy and Cell Biology, School of Dental Medicine, University of Pennsylvania, Philadelphia, PA 19104, USA,; 5 Department of Medicine, Section of Cardiology and; 6 Department of Pathology, The University of Chicago, Chicago, IL 60637, USA

## Abstract

Myostatin is a secreted signaling molecule that normally acts to limit muscle growth. As a result, there is extensive effort directed at developing drugs capable of targeting myostatin to treat patients with muscle loss. One potential concern with this therapeutic approach in patients with muscle degenerative diseases like muscular dystrophy is that inducing hypertrophy may increase stress on dystrophic fibers, thereby accelerating disease progression. To investigate this possibility, we examined the effect of blocking the myostatin pathway in *dysferlin*-deficient (*Dysf^−/−^*) mice, in which membrane repair is compromised, either by transgenic expression of follistatin in skeletal muscle or by systemic administration of the soluble form of the activin type IIB receptor (ACVR2B/Fc). Here, we show that myostatin inhibition by follistatin transgene expression in *Dysf^−/−^* mice results in early improvement in histopathology but ultimately exacerbates muscle degeneration; this effect was not observed in *dystrophin*-deficient (*mdx*) mice, suggesting that accelerated degeneration induced by follistatin transgene expression is specific to mice lacking *dysferlin. Dysf^−/−^* mice injected with ACVR2B/Fc showed significant increases in muscle mass and amelioration of fibrotic changes normally seen in 8-month-old *Dysf^−/−^* mice. Despite these potentially beneficial effects, ACVR2B/Fc treatment caused increases in serum CK levels in some *Dysf^−/−^* mice, indicating possible muscle damage induced by hypertrophy. These findings suggest that depending on the disease context, inducing muscle hypertrophy by myostatin blockade may have detrimental effects, which need to be weighed against the potential gains in muscle growth and decreased fibrosis.

## Introduction

Myostatin is a transforming growth factor-β (TGF-β) family member that acts as a negative regulator of skeletal muscle mass (1). Genetic studies in numerous species have shown that loss of myostatin results in dramatic increases in muscle mass (2–7), and pharmacological agents capable of blocking myostatin signaling have been shown to cause significant muscle hypertrophy when given systemically to mice (8). As a result, there has been considerable effort directed at developing myostatin inhibitors as potential therapeutic agents to increase muscle growth, and there are currently multiple such agents being tested in clinical trials in a wide range of indications characterized by muscle loss.

One indication for which myostatin inhibition is being tested as a potential therapy is muscular dystrophy. Muscular dystrophy encompasses a group of disorders characterized by progressive muscle degeneration and weakness (9). Many forms of muscular dystrophy result from inherited mutations in genes encoding proteins involved in maintaining the structural integrity of muscle fibers. The most common form affects dystrophin, which is a vital component of a protein complex that connects the cytoskeleton of a muscle fiber to the surrounding extracellular matrix; disruption of this link renders the muscle fibers susceptible to contraction-induced injury (10). In patients with severe forms of muscular dystrophy, the regenerative capacity of the muscle to respond to injury is unable to compensate for the degenerative process, leading to progressive loss of muscle function.

The identification of myostatin has raised the possibility that blocking this signaling pathway may be a therapeutic strategy to prevent or reverse the loss of muscle mass and strength in patients with muscular dystrophy, and numerous preclinical studies in *mdx* mice, which carry mutations in the *dystrophin* gene, have demonstrated beneficial effects of myostatin inhibition on muscle mass and function (11–18). One theoretical concern with this therapeutic approach is the possibility that inducing muscle hypertrophy may cause additional membrane stress to already fragile muscle fibers, thereby further inducing damage. If so, myostatin inhibition in patients with muscular dystrophy may provide short-term benefits in terms of increasing muscle growth but may have the long-term effect of accelerating disease progression. To investigate this issue, we examined the effect of blocking myostatin signaling in *dysferlin*-deficient (*Dysf^−/−^*) mice, which is a model for limb-girdle type 2B and Miyoshi muscular dystrophies. Our rationale for these studies was that, if myostatin inhibition results in increased membrane damage, these effects would be revealed specifically in *Dysf^−/−^* mice, in which membrane repair is compromised (19). Here, we show that inhibition of myostatin signaling indeed can result in an exacerbation of muscle degeneration in the setting of dysferlinopathy.

## Results

### Follistatin overexpression accelerates muscle loss in *Dysf^−/−^* mice

**Figure 1.**
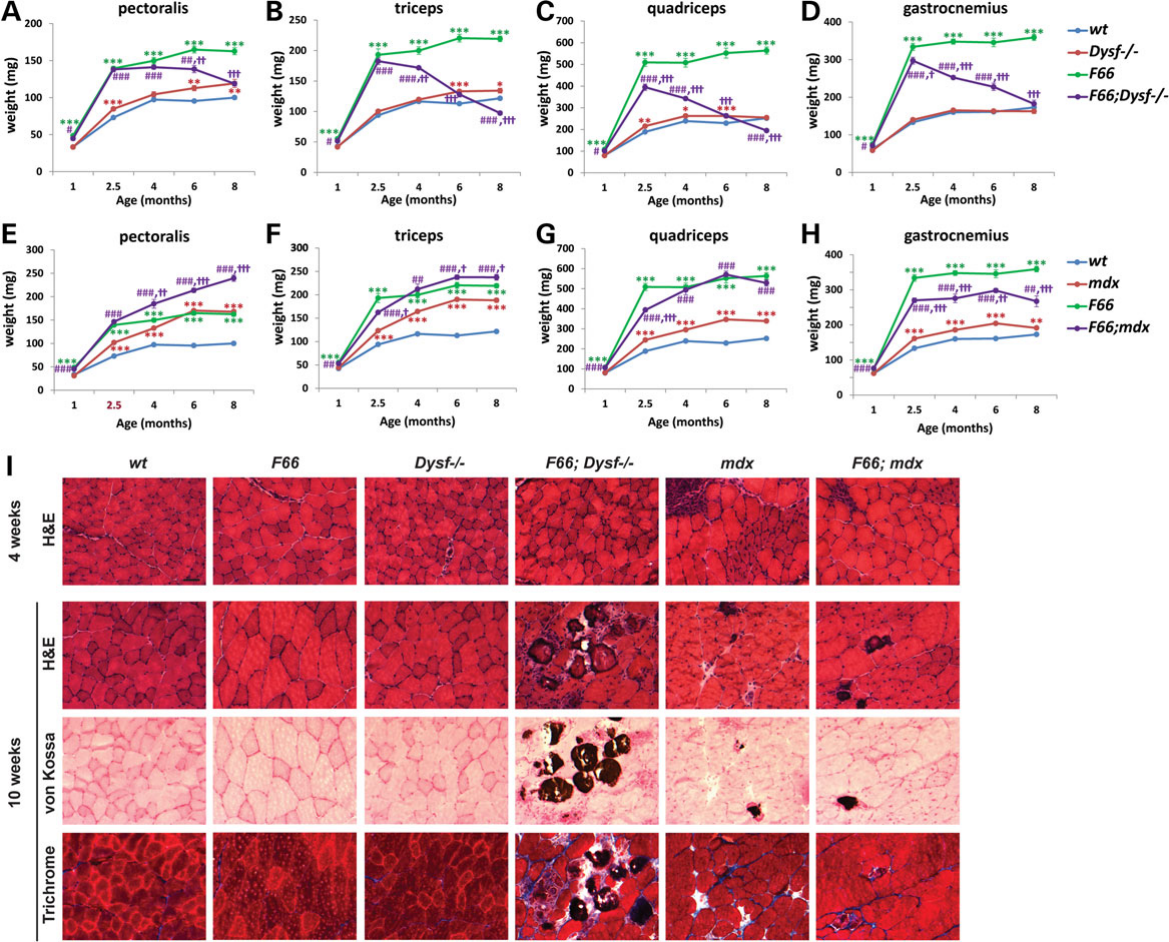
Hypertrophy induced by *Fst* transgene expression accelerates muscular degeneration in *Dysf^−/−^* mice. (**A**–**D**) Effect of *F66* on weights of pectoralis, triceps, quadriceps and gastrocnemius muscles in *Dysf^−/−^* mice as a function of age. Graphs were made from the data shown in Supplementary Material, Table S1. Note the dramatic loss of muscle mass induced by *F66* in *Dysf^−/−^* mice beginning at 4 months of age. *P < 0.05 versus *wt*; **P < 0.01 versus *wt*; ***P < 0.001 versus *wt*; ^#^*P* < 0.05 versus *Dysf^−/−^*; ^##^*P* < 0.01 versus *Dysf^−/−^*; ^###^*P* < 0.001 versus *Dysf^−/−^*; ^†^*P* < 0.05 versus *F66*; ^††^*P* < 0.01 versus *F66*; ^†††^*P* < 0.001 versus *F66*. (**E**–**H**) Effect of *F66* on weights of pectoralis, triceps, quadriceps and gastrocnemius muscles in *mdx* mice as a function of age. Note that *F66* did not induce muscle loss in *mdx* mice. Graphs were made from the data shown in Supplementary Material, Table S2. **P < 0.01 versus *wt*; ***P < 0.001 versus *wt*; ^##^*P* < 0.01 versus *mdx*; ^###^*P* < 0.001 versus *mdx*; ^†^*P* < 0.05 versus *F66*; ^††^*P* < 0.01 versus *F66*; ^†††^*P* < 0.001 versus *F66*. (**I**) Sections were stained with H&E, von Kossa (to highlight calcified fibers) and Masson's trichrome (to highlight areas of fibrosis). The *F66;Dysf^−/−^* mice showed the pathologic changes including fibrosis and centrally nucleated muscle fibers, even at 10 weeks of age, when *Dysf^−/−^* mice showed no distinct muscular degeneration. The *F66;mdx* mice showed the pathologic changes similar to *mdx* mice. Scale bar represents 50 µm.

To investigate the effect of blocking myostatin signaling in the setting of dysferlinopathy, we used both genetic and pharmacological approaches in mice. For the genetic approach, we overexpressed follistatin (Fst), which is a secreted protein capable of binding and inhibiting various members of the TGF-β superfamily, including myostatin (20). For these studies, we utilized a transgenic mouse line (*F66*) in which we had expressed Fst under the control of a skeletal muscle-specific myosin light chain promoter/enhancer (20). As shown in Figure 1A–D and Supplementary Material, Table S1, and as we reported previously, *F66* transgenic mice exhibit dramatic increases in skeletal muscle mass throughout the body. We examined the effect of crossing the *F66* transgene into *Dysf^−/−^* mice. At early ages, *F66* transgene expression in *Dysf^−/−^* mice (i.e. *F66;Dysf^−/−^* mice) caused increased muscle mass to a similar extent as *F66* did in a *wild-type* (*wt*) background (Fig. 1A–D; Supplementary Material, Table S1). Hence, blockade of the myostatin signaling pathway by Fst is capable of inducing muscle hypertrophy in the absence of functional dysferlin. These early gains in muscle weights, however, were not maintained as the mice aged, with muscle masses in *F66;Dysf^−/−^* mice decreasing sharply beginning at ∼4 months of age; in fact, by 8 months of age, *F66;Dysf^−/−^* mice showed even lower muscle weights than *Dysf^−/−^* mice.

A previous study using *mdx* mice had reported that gains in muscle mass due to loss of myostatin activity were maintained as the mice aged (21). Because that study had utilized the *Mstn* loss-of-function mutation to eliminate myostatin function, we sought to determine whether the effects that we had observed in *Dysf^−/−^* mice were somehow specific to the *F66* transgene by crossing the *F66* transgene onto an *mdx* background. In contrast to what we observed in *F66;Dysf^−/−^* mice, *F66;mdx* mice exhibited significantly increased muscle weights compared with *mdx* mice, which were maintained at all ages that we examined (Fig. 1E–H; Supplementary Material, Table S2). Hence, accelerated muscle loss induced by *Fst* transgene expression was specific to mice lacking dysferlin.

### Effect of Fst overexpression on muscle histopathology and function in *Dysf^−/−^* mice

The detrimental effect of *F66* in the absence of dysferlin was clearly seen upon histological examination of the muscles. As previously reported, histological analysis of muscles from *Dysf^−/−^* mice showed signs of a slowly progressive muscular degeneration (19); by 4 months of age, a few necrotic fibers and centrally located nuclei were detected, and by 8 months of age, most of pathological characteristics of muscular dystrophy were observed (Fig. 1I; Supplementary Material, Fig. S1). *F66;Dysf^−/−^* mice, however, showed more severe muscular degeneration than age-matched *Dysf^−/−^* mice. As early as 10 weeks of age, when increases in muscle weights due to the *F66* transgene were comparable in *wt* and *Dysf^−/−^* mice and when *Dysf^−/−^* muscles showed no distinct histological signs of degeneration, *F66;Dysf^−/−^* muscles showed dystrophic features, including central nucleation, fibrosis and calcified fibers (Fig. 1I). In contrast, although dystrophic changes were evident in muscles of *mdx* mice, the presence of the *F66* transgene did not exacerbate degeneration in the *mdx* background and even appeared to improve the histopathology as a function of age (Fig. 1I; Supplementary Material, Fig. S1); this improvement in histopathology resulting from *Fst* expression in *mdx* mice was also consistent with what had been reported in *mdx* mice lacking myostatin (11–15,17).

**Figure 2.**
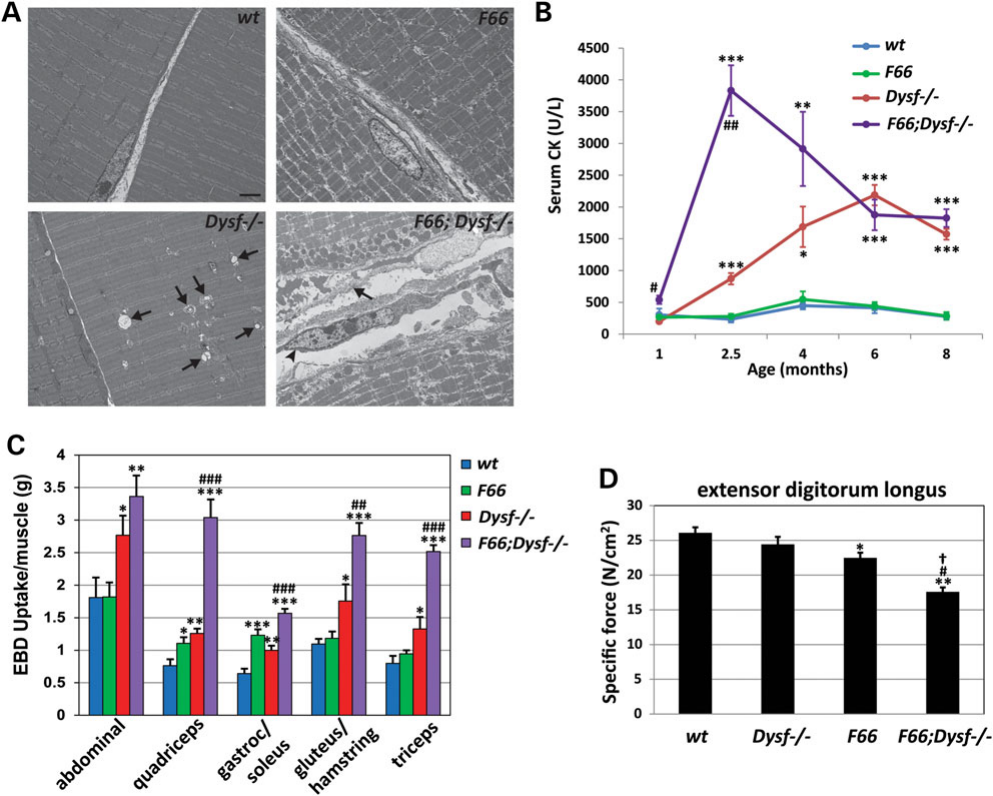
Effect of *Fst* transgene expression on muscle fiber integrity and function in *Dysf^−/−^* mice. (**A**) Electron microscope images of quadriceps muscles at 10 weeks of age. While *wt* mice showed the regular sarcomeric pattern with no degenerative change in mitochondrial morphology, *Dysf^−/−^* mice showed membrane-bound spaces surrounded by swollen mitochondria (arrows in *Dysf^−/−^* image). *F66* muscles showed randomly fragmented myofibrils in regular sarcomeric pattern, representing Type IIb fast-twitch fibers. *F66;Dysf^−/−^* muscles showed accumulation of vacuoles and mitochondria aggregation in subsarcolemmal area, necrotic fiber with complete loss of myofibrils (arrow in *F66;Dysf^−/−^* image) and fibroblast next to necrotic fiber (arrowhead). Scale bar represents 2 µm. (**B**) Serum CK (U/L) levels as a function of age. Note the sharp increase in CK levels in *F66;Dysf^−/−^* mice at 10 weeks of age. Graphs were made from the data shown in Supplementary Material, Table S3. *P < 0.05 versus *wt*; **P < 0.01 versus *wt*; ***P < 0.001 versus *wt*; ^#^*P* < 0.01 versus *Dysf^−/−^*; ^##^*P* < 0.001 versus *Dysf^−/−^*. (**C**) EBD uptake by abdominal, quadriceps, gastrocnemius/soleus, gluteus/hamstring and triceps muscles. Note the increased EBD uptake in *F66;Dysf^−/−^* mice compared with the other groups. Graphs were made from the data shown in Supplementary Material, Table S5. *P < 0.05 versus *wt*; **P < 0.01 versus *wt*; ***P < 0.001 versus *wt*; ^##^*P* < 0.01 versus *Dysf^−/−^*; ^###^*P* < 0.001 versus *Dysf^−/−^*. (**D**) Specific force of EDL muscles from *wt, Dysf^−/−^, F66* and *F66;Dysf^−/−^* mice at 4 months of age. *F66;Dysf^−/−^* mice showed a significant decrease in specific force compared with others. *P < 0.05 versus *wt*; **P < 0.01 versus *wt*; ^#^*P* < 0.05 versus *Dysf^−/−^*; ^†^*P* < 0.05 versus *F66*.

We also examined the muscles in *F66;Dysf^−/−^* mice by electron microscopy (EM). As shown in Figure 2A, EM analysis of quadriceps muscles of *Dysf^−/−^* mice at 10 weeks of age generally showed regular cross-striations with well-arranged myofibrils, although some fibers in *Dysf^−/−^* mice showed membrane-bound spaces surrounded by swollen mitochondria, and inflammatory cell infiltration between fibers was also seen. In *F66;Dysf^−/−^* mice, however, in addition to the inflammatory cell infiltration, mitochondrial degeneration was more prominent and severe, and necrotic fibers surrounded by fibroblasts were more frequently observed. These changes were not observed in *F66* muscles, which showed regular cross-striations with little evidence of muscle fiber degeneration, although we did observe occasional fibers in *F66* mice with randomly fragmented myofibrils.

The harmful effect of the *F66* transgene in *Dysf^−/−^* mice was also evident when we measured serum creatine kinase (CK, U/L) levels, which are routinely used as an indicator of muscle damage. As described previously (22), CK levels of *Dysf^−/−^* mice were elevated by 6 months of age, reflecting the ongoing muscle degeneration resulting from dysferlin deficiency (Fig. 2B; Supplementary Material, Table S3). *F66;Dysf^−/−^* mice, however, showed a sharp increase in CK levels at 10 weeks of age, which was observed neither in *F66* mice nor in *Dysf^−/−^* mice (*P* < 0.001 versus *Dysf^−/−^*). As mice aged, CK levels gradually declined in *F66;Dysf^−/−^* mice, presumably reflecting the loss of functional muscle as a result of the enhanced muscle degeneration. As reported previously (23), *mdx* mice had greatly elevated CK levels with high variability, and these elevations did not appear to be worsened by the presence of the *F66* transgene (Supplementary Material, Table S4).

We also evaluated the integrity of the plasma membrane by membrane-impermeable Evans blue dye (EBD) uptake. At 10 weeks of age, significant elevations in EBD uptake were observed in *Dysf^−/−^* mice compared with *wt* and *F66* mice in all muscle groups that were analyzed (Fig. 2C; Supplementary Material, Table S5), presumably resulting from the membrane repair defect in the absence of dysferlin. These elevations in EBD uptake, however, were exacerbated in *F66;Dysf^−/−^* mice, consistent with the elevated serum CK levels in these mice. Finally, we examined the effect of the *F66* transgene on muscle contractile function. Although no significant differences were seen at 10 weeks of age, *F66;Dysf^−/−^* mice showed a significant decrease in specific force of the EDL muscle (*P* < 0.01) at 4 months of age (Fig. 2D) compared with *wt*, *F66* and *Dysf^−/−^* mice. Hence, overexpression of Fst in *Dysf^−/−^* mice had a clear detrimental effect not only on muscle mass, histopathology and membrane integrity, but also on muscle contractile function.

### Effect of pharmacological blockade of myostatin signaling in *Dysf^−/−^* mice

**Figure 3.**
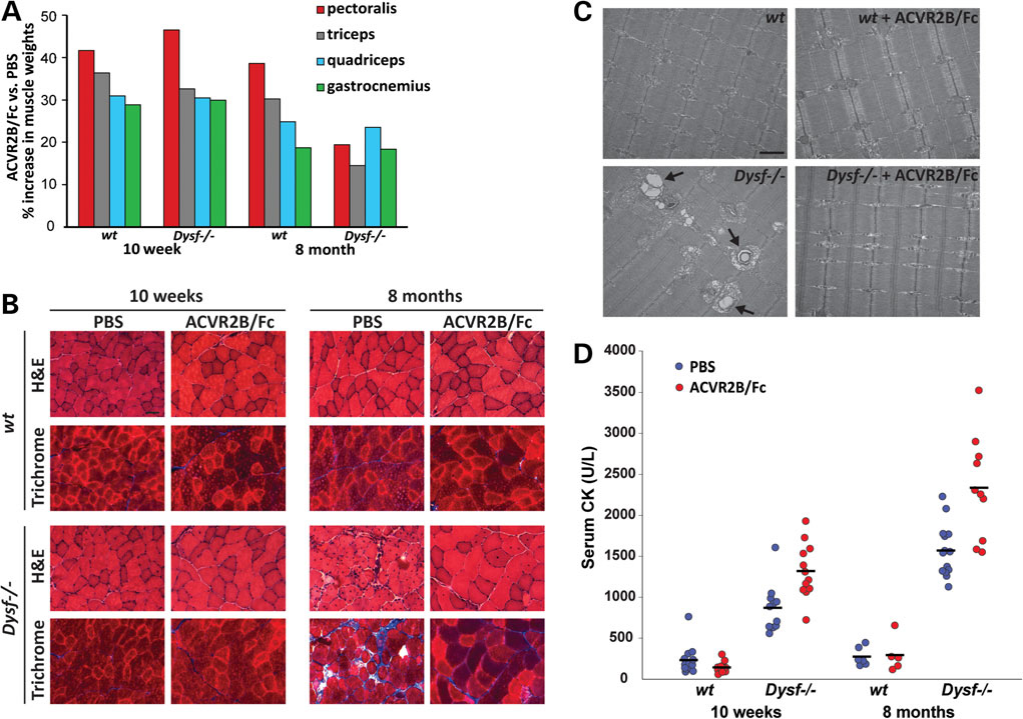
Effect of ACVR2B/Fc administration on muscle weights, muscle histopathology and serum CK levels in *Dysf^−/−^* mice. (**A**) Effect of ACVR2B/Fc on muscle weights. Bars indicate percent increase in muscle weights in ACVR2B/Fc-injected *wt* and *Dysf^−/−^* mice compared with PBS-injected *wt* and *Dysf^−/−^* mice, respectively. ACVR2B/Fc and PBS were administered by weekly i.p. injections over a span of 4 weeks at 10 mg kg^−1^ starting at age 6 weeks and 7 months. All calculations were made from the data shown in Supplementary Material, Table S6. (**B**) Sections of the quadriceps muscles stained with H&E and Masson's trichrome. Note that ACVR2B/Fc-injected *Dysf^−/−^* mice showed significant increases in muscle fiber diameters comparable to ACVR2B/Fc-injected *wt* mice and decreases in the extent of fibrosis compared with the PBS-injected *Dysf^−/−^* mice even at age 8 months. Scale bar represents 50 µm. (**C**) Electron microscope images. Note the membrane-bound spaces surrounded by swollen mitochondria in *Dysf^−/−^* myofiber (arrows). No specific changes were found in ACVR2B/Fc-injected *wt* muscles. However, ACVR2B/Fc-injected *Dysf^−/−^* myofiber showed reduction of membrane-bound spaces and swollen mitochondria compared with *Dysf^−/−^* myofiber. Scale bar represents 1 µm. (**D**) Effect of ACVR2B/Fc on serum CK levels. Note the increases in serum CK levels in ACVR2B/Fc-injected *Dysf^−/−^* mice compared with PBS-injected *Dysf^−/−^* mice at both 10 weeks and 8 months of age (*P* < 0.01). The graph was made from the data shown in Supplementary Material, Table S7.

As a complementary approach to these genetic studies, we also examined the effect of blocking myostatin signaling pharmacologically by direct administration of a myostatin inhibitor to *Dysf^−/−^* mice. We showed previously that myostatin is capable of binding the two activin type II receptors, ACVR2 and ACVR2B (20), and that a soluble form of ACVR2B in which the extracellular domain of the receptor is fused to an Fc domain can cause significant muscle hypertrophy when given systemically to *wt* mice (24). We examined the effect of administering this soluble receptor (ACVR2B/Fc) to *Dysf^−/−^* mice. As shown in Figure 3A and Supplementary Material, Table S6, *Dysf^−/−^* mice given four weekly intraperitoneal (i.p.) injections of ACVR2B/Fc beginning at either 6 weeks or 7 months of age showed significant increases in muscle mass comparable to *wt* mice injected with ACVR2B/Fc. Moreover, fibrotic changes seen in 8-month-old *Dysf^−/−^* mice appeared to be significantly ameliorated by ACVR2B/Fc treatment (Fig. 3B); these histological improvements were consistent with what had been reported for ACVR2B/Fc treatment of *mdx* mice (18). EM analysis also showed that membrane-bound spaces surrounded by swollen mitochondria normally seen in *Dysf^−/−^* myofibers were less prominent in muscles of ACVR2B/Fc-treated *Dysf^−/−^* mice (Fig. 3C). Despite these potentially beneficial effects, ACVR2B/Fc treatment caused increases in serum CK levels in some *Dysf^−/−^* mice at both 10 weeks and 8 months of age (Fig. 3D; Supplementary Material, Table S7), suggesting that hypertrophy induced by ACVR2B/Fc administration to *Dysf^−/−^* mice may have some adverse effects as well, though not nearly to the degree seen with *Fst* overexpression.

### Effect of myostatin blockade on global gene expression patterns in *wt* and *Dysf^−/−^* mice

**Figure 4.**
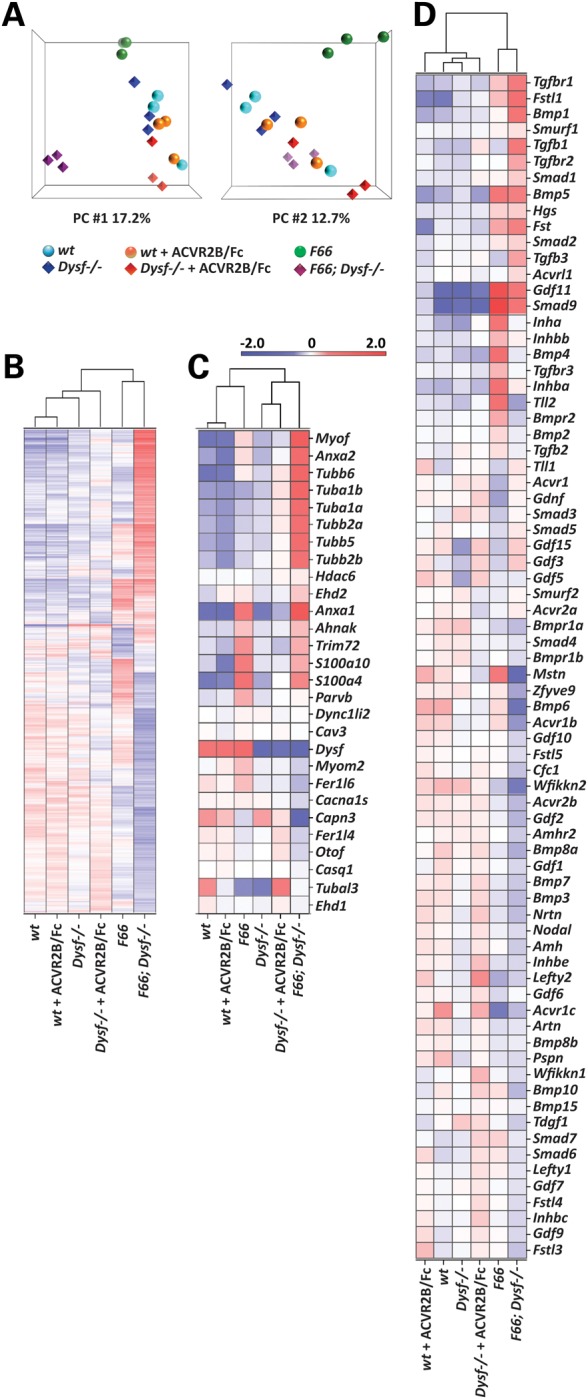
Analysis of global gene expression patterns in *wt, Dysf^−/−^, F66*, *F66; Dysf^−/−^* and ACVR2B/Fc-injected *wt* and *Dysf^−/−^* mice. (**A**) PCA mapping analysis. Affymetrix Mouse Exon 1.0 ST arrays were hybridized in three biologically independent experiments with RNA from quadriceps muscles of *wt* (blue circle)*, Dysf^−/−^* (blue diamond)*, F66* (green circle), *F66;Dysf^−/−^* (purple diamond) mice and ACVR2B/Fc-injected *wt* (orange circle) and *Dysf^−/−^* (red diamond) mice (three replicates in six different groups: 18 samples). Note that *F66;Dysf^−/−^* and *F66* groups are well segregated from each other and from the other remaining groups in PCA mappings. First component (PC # 1) and second component (PC # 2) data are shown. (**B**) Heat map corresponding to differentially expressed genes (*P* < 0.01). Data represent averages of three independent experiments in red (enriched) or blue (depleted). (**C**) Heat map corresponding to genes related to dysferlin function*.* Relative expression levels (fold change compared to *wt*) are shown in Supplementary Material, Table S8. (**D**) Heat map corresponding to genes encoding components of the myostatin/TGF-β signaling pathway. Relative expression levels (fold change compared to *wt*) are shown in Supplementary Material, Table S8.

Finally, we used microarray analysis to examine global transcriptional changes in the quadriceps muscles from *wt, Dysf^−/−^, F66*, *F66;Dysf^−/−^* mice and ACVR2B/Fc-injected *wt* and *Dysf^−/−^* mice at 10 weeks of age. By principal components analysis (PCA) mapping, the *F66;Dysf^−/−^* and *F66* groups were well segregated from each other as well as the other groups (Fig. 4A). The differences of global gene expression patterns among the six groups were also seen in their heat maps (Fig. 4B). As expected, two classes of genes that showed clear differences among the various groups were genes related to dysferlin function and genes encoding components of the myostatin/TGF-β signaling pathway (Fig. 4C and D). Seven genes related to dysferlin function, *Dysf, Myof, Anxa1, Anxa2, S100a4, S100a10* and *Capn3*, were visualized in Volcano plots in five different comparisons (Supplementary Material, Fig. S2); *Myof, Anxa1, Anxa2, S100a4* and *S100a10* were significantly upregulated by *Fst* overexpression while *Capn3* was significantly downregulated by *Fst* overexpression. *Mstn*, *Gdf11*, *Inha* and *Inhba* genes encode proteins that have been shown to be bound and inhibited by Fst and ACVR2B/Fc. While there was no significant change in *Dysf^−/−^* compared with *wt*, *Fst* overexpression and ACVR2B/Fc administration induced the dramatic changes of expression levels of these genes in both *Dysf^−/−^* and *wt* (Supplementary Material, Fig. S3); *Gdf11* was upregulated slightly in ACVR2B/Fc-injected *wt* and ACVR2B/Fc-injected *Dysf^−/−^* and significantly in *F66* and *F66;Dysf^−/−^*; *Mstn* was downregulated slightly in ACVR2B/Fc-injected *Dysf^−/−^* compared with *Dysf^−/−^* and significantly in *F66;Dysf^−/−^* compared with *Dysf^−/−^*. The full list of genes with fold changes is given in Supplementary Material, Table S8.

**Figure 5.**
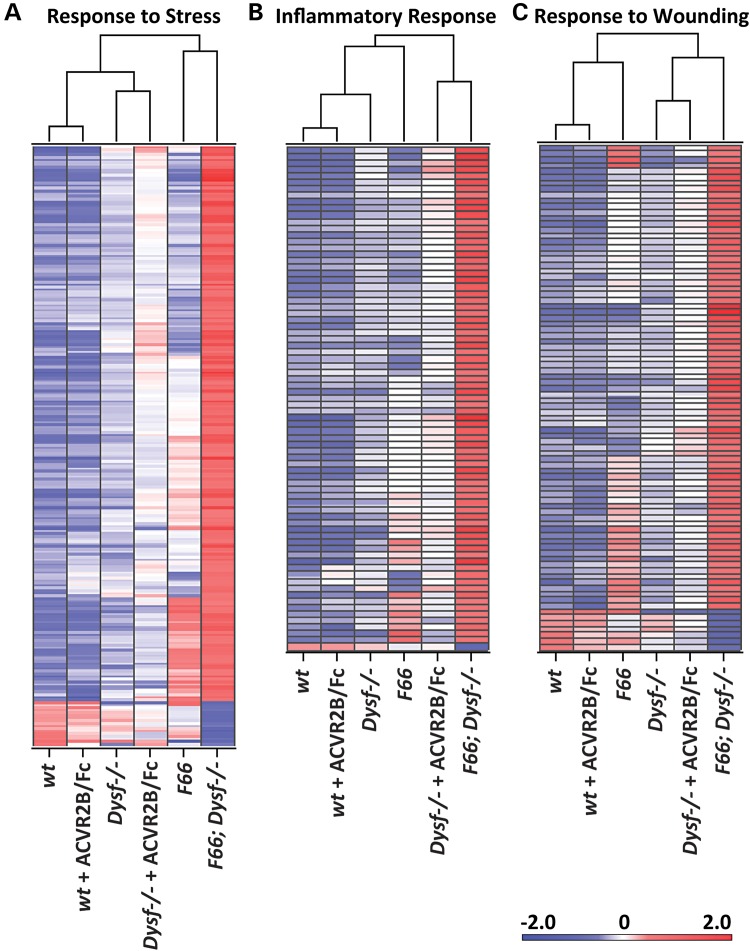
Heat maps of enriched top biological processes from GO analysis. (**A**) Heat map corresponding to response to stress (205 genes). Note that *F66* and *F66;Dysf^−/−^* are grouped together. (**B**) Heat map corresponding to inflammatory response (73 genes). Note that ACVR2B/Fc-injected *Dysf^−/−^* and *F66;Dysf^−/−^* are grouped together. (**C**) Heat map corresponding to response to wounding (73 genes). Note that *Dysf^−/−^*, ACVR2B/Fc-injected *Dysf^−/−^* and *F66;Dysf^−/−^* are grouped together. Data represent averages of three independent experiments in red (enriched) or blue (depleted). GO analysis was conducted for significantly differentially expressed genes (fold change >2.0 and *P* < 0.01), and enriched top biological processes are shown in Supplementary Material, Table S9. List of genes in each biological process with relative expression levels (fold change compared with *wt*) is shown in Supplementary Material, Dataset.

Biological processes altered by either *Fst* overexpression or ACVR2B/Fc treatment were detected by Gene ontology (GO) analysis (Supplementary Material, Table S9). The differential expression patterns of genes involved in processes related to response to stress were observed and shown in their heat maps (Fig. 5; for additional details, see Supplementary Material, Dataset). *Fst* overexpression in both *wt* and *Dysf^−/−^* background dramatically changed the expression pattern of genes related to response to stress (Fig. 5A). Hypertrophy induced by either *Fst* overexpression or ACVR2B/Fc treatment in *Dysf^−/−^* background resulted in similar expression patterns of genes related to inflammatory responses (Fig. 5B) and response to wounding (Fig. 5C), consistent with the important membrane repair function of dysferlin. Hence, the differences that we observed on muscle mass and histopathology among the various experimental groups were clearly reflected in the differential expression patterns of genes related to altered biological processes.

## Discussion

The identification of myostatin as a negative regulator of skeletal muscle mass has generated considerable interest in the possibility that targeting the myostatin pathway may be an effective strategy to enhance muscle growth and function in patients with muscle loss. Indeed, the potential benefits of myostatin inhibition have been documented in numerous models of muscle wasting and degeneration in mice (for review, see ref. 8), and there are now several studies that have reported that myostatin inhibition can increase lean body mass and improve muscle function in humans as well (25–27). In this respect, a number of myostatin inhibitors are currently being tested in Phase II/III clinical trials for a variety of indications characterized by muscle loss, including cancer, hip replacement surgery, age-related sarcopenia, end-stage renal disease, chronic obstructive pulmonary disease and inclusion body myositis (see clinicaltrials.gov).

Although increasing muscle growth in these disease settings may provide short-term benefit, there are potential long-term concerns about the use of this therapeutic strategy. One theoretical concern is that inducing muscle hypertrophy may promote muscle damage by increasing stress on myofibers. Although this effect may be inconsequential under normal conditions, the increased damage to fibers might accelerate disease progression when muscle fibers are already fragile, for example, as a result of genetic loss of key structural components in the case of muscular dystrophy. Here, we have presented results of studies in which we have attempted to address this possibility by analyzing the effect of blocking myostatin signaling in mice lacking dysferlin. Because studies have shown that dysferlin is likely involved in Ca^2+^-dependent membrane repair of damaged skeletal muscle fibers (19,28,29), we reasoned that potential detrimental effects of myostatin inhibition in terms of increasing muscle fiber stress and damage might be more pronounced in *Dysf^−/−^* mice. Our results show that myostatin inhibition does, in fact, accelerate muscle degeneration in *Dysf^−/−^* mice and suggest that the anabolic effects of myostatin inhibition may come at the expense of additional damage to muscle fibers.

It will be important to weigh these potential adverse effects against the beneficial effects seen with myostatin inhibition not only on muscle growth but also on histopathology, such as decreased fibrosis. In mice in which myostatin signaling was blocked by Fst transgene expression, the massive initial muscle hypertrophy was clearly outweighed by the subsequent detrimental effects leading to accelerated muscle degeneration. In mice treated with ACVR2B/Fc, however, the beneficial effects on muscle mass and histopathology were significant, with the only evidence of adverse effects being the elevated CK levels. In this regard, the beneficial effects of ACVR2B/Fc administration to *Dysf^−/−^* mice were observed even in mice at 14 months of age (Supplementary Material, Table S6). Further studies using a variety of different myostatin inhibitors, treatment timing/durations, and doses will be essential in determining whether the beneficial effects of myostatin inhibition can be achieved while minimizing the potential detrimental effects. It will also be critical to conduct these types of studies not only in the setting of chronic muscle degenerative diseases but also in clinical settings characterized by more acute muscle loss, in which the fragility and structural integrity of the muscle fibers may not be as important an issue.

## Materials and Methods

### Mice

*F66* transgenic mice that express *Fst* under the myosin light chain promoter have been described previously (20). Because the *F66* transgene is located on the Y chromosome, we focused all of our analysis on male mice. To analyze the effect of *F66* in *Dysf^−/−^* mice, *F66* transgenic mice were mated with *Dysf^−/−^* mice. *F66;Dysf^+/−^* males from this cross were mated to *Dysf^+/−^* females obtain *F66;Dysf^−/−^* and *F66;Dysf^+/+^* males. As the *mdx* mutation in *mdx* mouse is X chromosome-linked, *F66;mdx* male mice were generated from matings between *F66* males and *mdx* females. All mice were maintained on a C57BL/6 background.

### ACVR2B/Fc purification and injection

ACVR2B/Fc is a soluble form of the activin type IIB receptor (ACVR2B) in which the extracellular ligand binding domain was fused to the mouse IgG2a Fc domain (ACVR2B/Fc) to enhance stability *in vivo* (24). ACVR2B/Fc protein expression from the stable Chinese hamster ovary (CHO) cells was induced with 100 nm Cadmium in serum-free EX-CELL™ 325 PF CHO media (Sigma, St Louis, MO, USA), and ACVR2B/Fc protein was purified from the conditioned medium using protein A Sepharose. Male C57BL/6 (*wt*) and *Dysf^−/−^* mice beginning at either 6 weeks or 7 months of age were given four weekly intraperitoneal (i.p.) injections of either ACVR2B/Fc (10 mg kg^−1^, i.e. 200 μg per injection for 6-week-old mice and 250 μg per injection for 7-month-old mice) or PBS.

### Muscle weight and histopathologic analysis

For measurement of muscle weights, pectoralis, triceps, quadriceps and gastrocnemius muscles from both sides of mice aged from 4 weeks to 8 months were dissected, and the average weight was used for each muscle. For histopathologic analysis, the harvested quadriceps muscles were snap frozen in isopentene cooled in liquid nitrogen, and 10 µm frozen cross-sections were taken from the right quadriceps and subjected to hematoxylin/eosin (H&E), von Kossa (to highlight calcified fibers) and Masson's trichrome staining (to highlight areas of fibrosis). Images were acquired with a ×20 objective using a Zeiss AxioCam MRc5 microscope in combination with AxioVision 4.8 software.

### Evans blue dye uptake

To detect plasma membrane disruption, 10 µl per 1 g of body weight of sterile EBD (5 mg/ml in PBS) was injected intraperitoneally into 10-week-old mice 2 days before harvesting. Abdominal, quadriceps, gastrocnemius/soleus, gluteus/hamstring and triceps muscles were dissected, minced and incubated in formamide at 55°C for 2 h, and the supernatant absorbances were measured at 620 nm in a spectrophotometer.

### Isolated muscle mechanics

Mice were anesthetized with ketamine/xylazine, and muscles were removed and prepared as previously described (30). Isolated muscle mechanics, including isometric tetanic force as well as susceptibility to eccentric contractile injury, were performed on the extensor digitorum longus (EDL). Specific force was measured as force per cross-section area (*n* = 3 per age and genotype).

### Transmission electron microscopy

Quadriceps muscles of 10-week-old mice were fixed with 2% glutaraldehyde and 2% formaldehyde in 0.1 m cacodylate buffer (pH 7.4), and post-fixed with 1% osmium tetroxide in 0.1 m cacodylate buffer. Fixed samples were en bloc stained with 2% uranyl acetate, dehydrated through series of ethanol and propylene oxide, and embedded in EMbed-812 resin. Ultrathin sections were cut and stained with uranyl acetate and lead citrate. Photographs were taken through the Philips BioTwin CM120 electron microscope.

### Microarray analysis

Total RNA was extracted from quadriceps muscles of 10-week-old *wt, Dysf^−/−^, F66*, *F66; Dysf^−/−^*, and ACVR2B/Fc-injected *wt* and *Dysf^−/−^* mice (six different groups), and three biological replicates were set up for each group. Affymetrix Mouse Exon 1.0 ST arrays were hybridized with extracted RNA (three replicates in six different groups, 18 samples) at the Johns Hopkins Microarray Core Facility. Microarray data were visualized by PCA mapping, volcano plots and heat maps. GO (www.geneontology.org) analysis was conducted for significantly differentially expressed genes (fold change >2.0 and *P* < 0.01) using the Spotfire analytic platform (31). Raw microarray data have been deposited in the Gene Expression Omnibus database (GSE62945).

### Statistical analysis

All values are presented as mean ± SEM from at least three independent experiments unless otherwise stated. An unpaired two-tailed Student's *t-*test and one-way ANOVA were performed for statistical analyses with two groups and more than two groups, respectively. *P* < 0.05 was considered significant.

### Study approval

Mice were handled and housed according to the approved Institutional Animal Care and Use Committee (IACUC) protocols MO12M07 and MO13M283 of the Johns Hopkins Medical Institutions. All animal studies were approved by the IACUC of the Johns Hopkins Medical Institutions.

## Supplementary Material

Supplementary material is available at *HMG* online.

## Funding

This work was supported by the National Institutes of Health (R01AR059685 to S.-J.L., R01AR060636 to S.-J.L., P01NS0720027 to E.M.M.) and the Jain Foundation (to S.-J.L.). S.-J.L. was supported by Lawrence Ellison Foundation Senior Scholar in Aging Award AG-SS-2678-11 and by generous gifts from Michael and Ann Hankin, Partners of Brown Advisory, and James and Julieta Higgins. Funding to pay the Open Access publication charges for this article was provided by Lawrence Ellison Foundation Senior Scholar in Aging Award AG-SS-2678-11.

## Supplementary Material

Supplementary Data
