# Supplementary material for: Muscle hypertrophy induced by myostatin inhibition accelerates degeneration in dysferlinopathy
Source: Hum Mol Genet. 2015 Jul 23;24(20):5711–9. doi: 10.1093/hmg/ddv288 (PMC4581601; doi:10.1093/hmg/ddv288)
Supplement: Supplementary Data [file supp_24_20_5711__index.html]

Muscle hypertrophy induced by myostatin inhibition accelerates degeneration in dysferlinopathy — Muscle hypertrophy induced by myostatin inhibition accelerates degeneration in dysferlinopathy — Supplementary Data 

# Muscle hypertrophy induced by myostatin inhibition accelerates degeneration in dysferlinopathy

## Supplementary Data

Supplementary Data

- Supplementary Data - Docx file
- Supplementary Dataset - xlsx file
