# Supplementary material for: Muscle hypertrophy induced by myostatin inhibition accelerates degeneration in dysferlinopathy
Source: Hum Mol Genet. 2015 Jul 23;24(20):5711–9. doi: 10.1093/hmg/ddv288 (PMC4581601; doi:10.1093/hmg/ddv288)
Supplement: Supplementary Data [file supp_ddv288_ddv288supp.docx]

**Supplementary Material**

**Supplementary Figures**

**
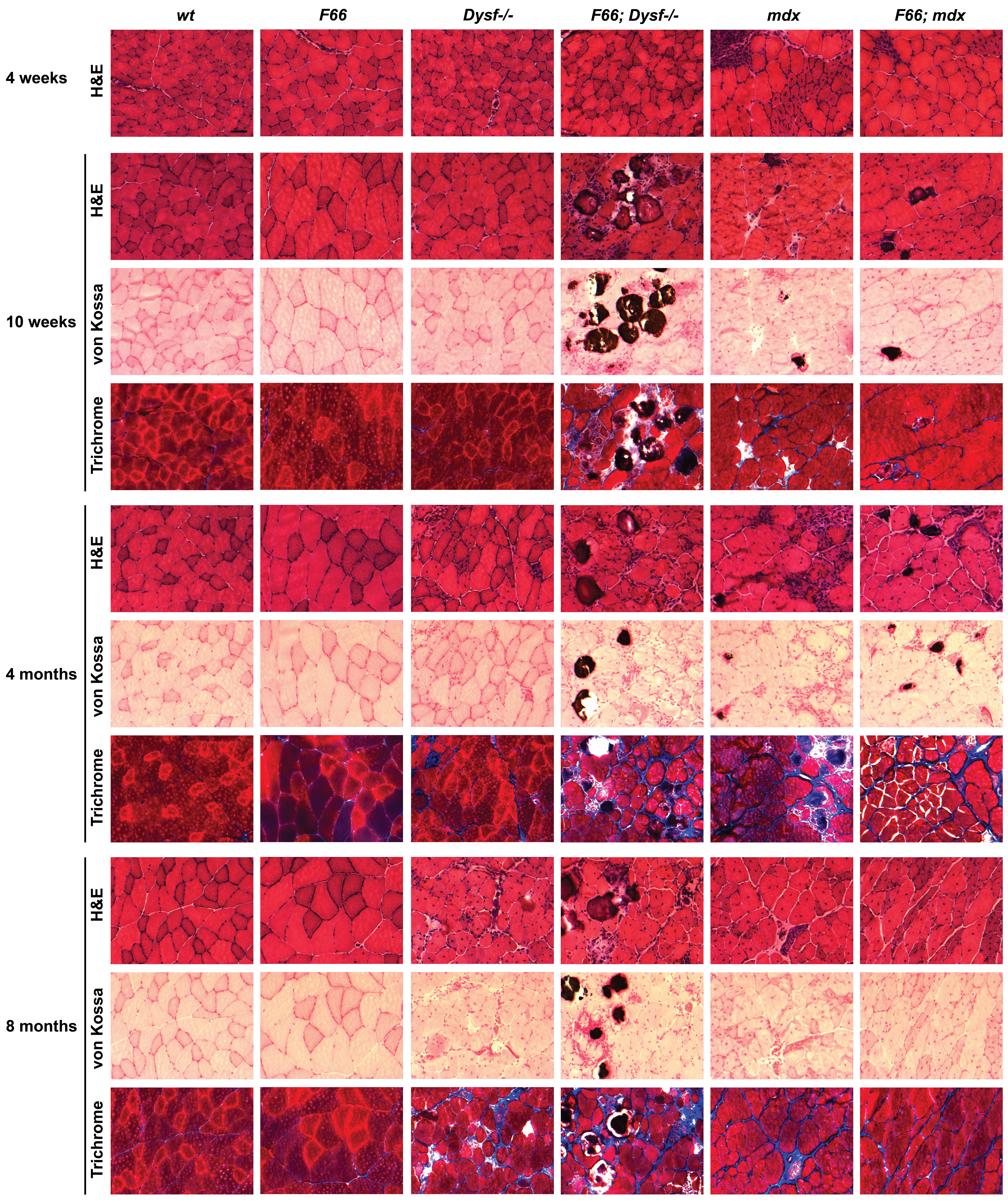
**

**Figure S1.** Histopathologic analysis of *Dysf^-/-^* mice carrying the *Fst* transgene. Panels show serial sections of the quadriceps muscles isolated from *wt*, *F66*, *Dysf^-/-^*, *F66;Dysf^-/-^*, *mdx*, and *F66;mdx* mice aged from 4 weeks to 8 months. Sections were stained with H&E, von Kossa (to highlight calcified fibers), and Masson’s trichrome (to highlight areas of fibrosis). The *F66;Dysf^-/-^* mice showed the pathologic changes including fibrosis and centrally nucleated muscle fibers, even at 10 weeks of age, when *Dysf^-/-^* mice showed no distinct muscular degeneration. The *F66;mdx* mice showed the pathologic changes similar to *mdx* mice. Scale bar represents 50 µm.

**
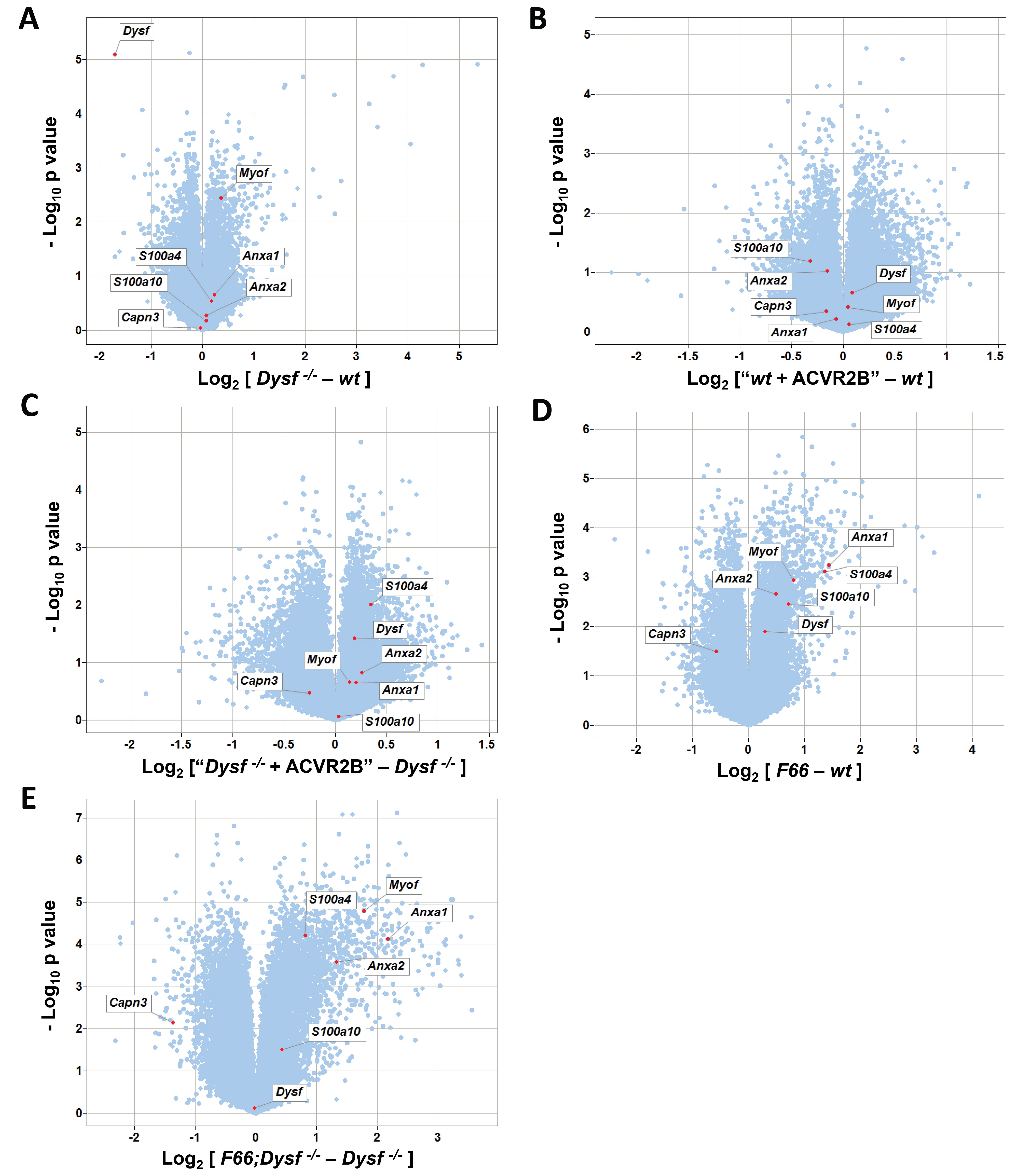
**

**Figure S2.** Differential expressions of genes related to dysferlin function. Seven genes, *Dysf,* *Myof, Anxa1, Anxa2, S100a4, S100a10,* and *Capn3*, were visualized in Volcano plots in 5 different comparisons; (A) *wt* versus *Dysf^-/-^*, (B) *wt* versus ACVR2B/Fc-injected *wt*, (C) *Dysf^-/-^* versus ACVR2B/Fc-injected *Dysf^-/-^*, (D) *wt* versus *F66*, (E) *Dysf^-/-^* versus *F66;Dysf^-/-^*. Note that only *Capn3* was down-regulated by *Fst* overexpression. The full list of genes with fold changes is given in Table S8.

**
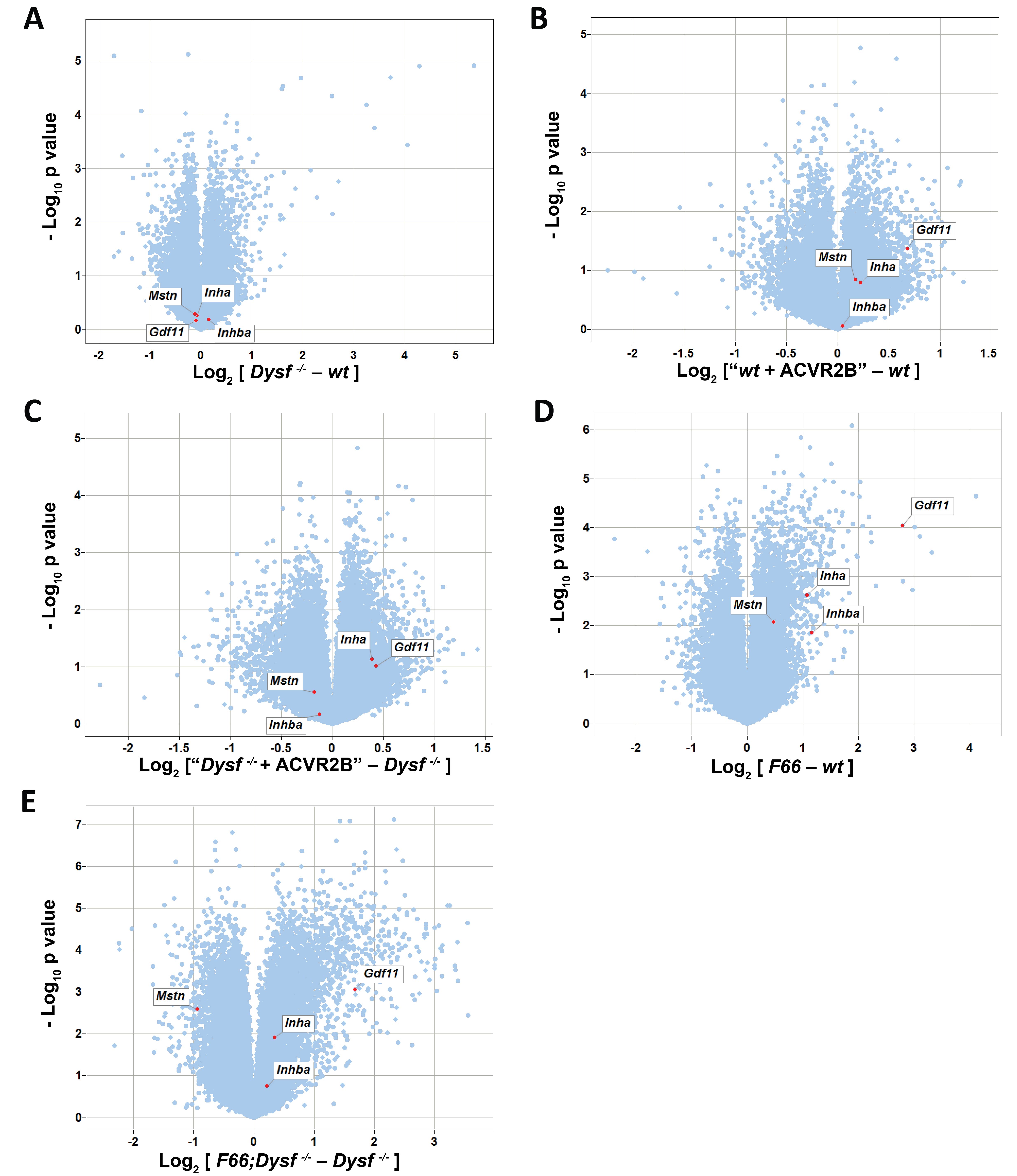
**

**Figure S3.** Differential expressions of *Mstn*, *Gdf11*, *Inha*, and *Inhba*. These genes encode proteins which have been shown to be bound and inhibited by Fst and ACVR2B/Fc. While there was no significant change in *Dysf^-/-^* compared to *wt* (A), *Fst* overexpression and ACVR2B/Fc administration induced the dramatic changes of expression levels of these genes (B-E). *Mstn* was down-regulated slightly in ACVR2B/Fc-injected *Dysf^-/-^* compared to *Dysf^-/-^* (C), and significantly in *F66;Dysf^-/-^* compared to *Dysf^-/-^* (E). *Gdf11* was up-regulated slightly in ACVR2B/Fc-injected *wt* (B) and ACVR2B/Fc-injected *Dysf^-/-^* (C), and significantly in *F66* (D) and *F66;Dysf^-/-^* (E). The full list of genes with fold changes is given in Table S8.

**Supplementary Tables**

**Table S1.** Effect of the follistatin transgene (*F66*) on muscle weights (mg) in *wt* and *Dysf^-/-^* mice at the ages from 4 weeks to 8 months.

|  |  | | | muscle weights (mg) | | | | |
| --- | --- | --- | --- | --- | --- | --- | --- | --- |
|  |  | body weight (g) | | pectoralis | triceps | quadriceps | gastrocnemius | |
| ***wt*** |  | |  |  |  |  | |  |
| 4 week | (n = 14) | | 14.1 ± 0.5 | 33.5 ± 1.4 | 42.5 ± 1.3 | 80.1 ± 2.7 | | 61.8 ± 1.9 |
| 10 week | (n = 10) | | 23.7 ± 0.5 | 73.2 ± 1.5 | 93.8 ± 1.7 | 188.7 ± 3.8 | | 133.3 ± 2.1 |
| 4 month | (n = 8) | | 28.6 ± 0.8 | 97.4 ± 1.3 | 116.4 ± 2.1 | 238.9 ± 5.5 | | 159.9 ± 2.7 |
| 6 month | (n = 7) | | 36.2 ± 1.3 | 95.5 ± 1.6 | 112.9 ± 1.2 | 229.1 ± 3.3 | | 161.2 ± 2.6 |
| 8 month | (n = 8) | | 39.1 ± 1.3 | 100.1 ± 2.3 | 121.6 ± 2.5 | 252.0 ± 5.8 | | 172.9 ± 3.5 |
| ***Dysf ^-/-^*** |  | |  |  |  |  | |  |
| 4 week | (n = 5) | | 14.1 ± 0.8 | 33.2 ± 2.4 | 41.8 ± 2.0 | 79.7 ± 4.6 | | 58.4 ± 2.9 |
| 10 week | (n = 8) | | 25.3 ± 0.8 | 84.9 ± 2.4 **^c^** | 100.2 ± 2.8 | 215.0 ± 7.3 **^b^** | | 139.9 ± 4.5 |
| 4 month | (n = 7) | | 29.9 ± 0.9 | 104.4 ± 3.6 | 119.6 ± 3.1 | 262.4 ± 8.0 **^a^** | | 165.2 ± 4.6 |
| 6 month | (n = 8) | | 30.7 ± 0.7 **^b^** | 112.9 ± 3.5 **^b^** | 132.9 ± 2.9 **^c^** | 262.0 ± 5.8 **^c^** | | 163.2 ± 4.3 |
| 8 month | (n = 7) | | 32.3 ± 1.3 **^b^** | 119.2 ± 5.5 **^b^** | 134.1 ± 4.0 **^a^** | 254.6 ± 7.4 | | 162.6 ± 6.1 |
|  |  | |  |  |  |  | |  |
| ***F66*** |  | |  |  |  |  | |  |
| 4 week | (n = 11) | | 15.0 ± 0.4 | 48.6 ± 1.7 **^c^** | 55.2 ± 1.4 **^c^** | 107.9 ± 3.3 **^c^** | | 75.3 ± 2.0 **^c^** |
| 10 week | (n = 7) | | 31.8 ± 0.5 **^c^** | 139.4 ± 3.0 **^c^** | 193.1 ± 9.6 **^c^** | 509.1 ± 17.8**^c^** | | 333.9 ± 9.5 **^c^** |
| 4 month | (n = 11) | | 35.7 ± 0.8 **^c^** | 149.9 ± 4.4 **^c^** | 199.9 ± 6.2 **^c^** | 507.7 ± 20.1**^c^** | | 347.9 ± 6.9 **^c^** |
| 6 month | (n = 9) | | 37.9 ± 0.8 | 164.9 ± 4.6 **^c^** | 220.3 ± 6.3 **^c^** | 552.3 ± 22.9**^c^** | | 345.7 ±11.3**^c^** |
| 8 month | (n = 9) | | 38.3 ± 1.0 | 162.7 ± 4.7 **^c^** | 219.2 ± 4.5 **^c^** | 563.7 ± 16.2**^c^** | | 359.0 ± 7.9 **^c^** |
|  |  | |  |  |  |  | |  |
| ***F66; Dysf ^-/-^*** | | |  |  |  |  | |  |
| 4 week | (n = 10) | | 14.5 ± 0.7 | 44.8 ± 2.7 **^d^** | 51.6 ± 2.6 **^d^** | 101.1 ± 5.7 **^d^** | | 71.6 ± 3.5 **^d^** |
| 10 week | (n = 11) | | 30.7 ± 0.7 **^f^** | 138.1 ± 3.0 **^f^** | 182.5 ± 5.5 **^f^** | 394.8 ±14.6 **^f,i^** | | 297.2 ± 9.2 **^f,g^** |
| 4 month | (n = 9) | | 34.0 ± 0.6 **^e^** | 141.2 ± 2.8 **^f^** | 171.7 ± 2.6 **^f,h^** | 342.6 ± 7.1 **^f,i^** | | 252.3 ± 4.2 **^f,i^** |
| 6 month | (n = 8) | | 34.7 ± 0.6 **^f,h^** | 138.6 ± 4.9 **^e,h^** | 128.0 ± 4.2 **^i^** | 262.9 ± 9.6 **^i^** | | 227.4 ± 9.6 **^f,i^** |
| 8 month | (n = 9) | | 35.5 ± 1.0 | 118.8 ± 3.1 **^i^** | 97.3 ± 2.8 **^f,i^** | 194.9 ± 6.0 **^f,i^** | | 182.4 ± 9.6 **^i^** |
|  |  | |  |  |  |  | |  |

Comparison between age-matched groups. **^a^** *p <* 0.05 vs. *wt*; **^b^** *p <* 0.01 vs. *wt*; **^c^** *p <* 0.001 vs. *wt*; **^d^** *p* < 0.05 vs. *Dysf ^-/-^*; **^e^** *p* < 0.01 vs. *Dysf ^-/-^*; **^f^** *p* < 0.001 vs. *Dysf ^-/-^*; **^g^** *p <* 0.05 vs. *F66*; **^h^** *p <* 0.01 vs. *F66*; **^i^** *p <* 0.001 vs. *F66*. Mean ± SEM.

**Table S2.** Effect of the follistatin transgene (*F66*) on muscle weights (mg) in *wt* and *mdx* mice at the ages from 4 weeks to 8 months.

|  |  | | | muscle weights (mg) | | | | |
| --- | --- | --- | --- | --- | --- | --- | --- | --- |
|  |  | body weight (g) | | pectoralis | triceps | quadriceps | gastrocnemius | |
| ***wt*** |  | |  |  |  |  | |  |
| 4 week | (n = 14) | | 14.1 ± 0.5 | 33.5 ± 1.4 | 42.5 ± 1.3 | 80.1 ± 2.7 | | 61.8 ± 1.9 |
| 10 week | (n = 10) | | 23.7 ± 0.5 | 73.2 ± 1.5 | 93.8 ± 1.7 | 188.7 ± 3.8 | | 133.3 ± 2.1 |
| 4 month | (n = 8) | | 28.6 ± 0.8 | 97.4 ± 1.3 | 116.4 ± 2.1 | 238.9 ± 5.5 | | 159.9 ± 2.7 |
| 6 month | (n = 7) | | 36.2 ± 1.3 | 95.5 ± 1.6 | 112.9 ± 1.2 | 229.1 ± 3.3 | | 161.2 ± 2.6 |
| 8 month | (n = 8) | | 39.1 ± 1.3 | 100.1 ± 2.3 | 121.6 ± 2.5 | 252.0 ± 5.8 | | 172.9 ± 3.5 |
| ***mdx*** |  | |  |  |  |  | |  |
| 4 week | (n = 9) | | 12.9 ± 0.4 | 30.9 ± 1.1 | 44.5 ± 1.7 | 81.1 ± 2.8 | | 61.7 ± 2.3 |
| 10 week | (n = 11) | | 26.1 ± 0.4 **^a^** | 102.0 ± 1.9 **^b^** | 123.3 ± 2.3 **^b^** | 244.5 ± 4.5 **^b^** | | 161.0 ± 2.9 **^b^** |
| 4 month | (n = 11) | | 28.7 ± 0.5 | 132.9 ± 2.5 **^b^** | 164.3 ± 2.8 **^b^** | 295.4 ± 5.0 **^b^** | | 185.7 ± 4.7 **^b^** |
| 6 month | (n = 10) | | 33.5 ± 0.5 | 170.3 ± 2.7 **^b^** | 190.1 ± 3.4 **^b^** | 346.8 ± 3.7 **^b^** | | 204.4 ± 3.2 **^b^** |
| 8 month | (n = 11) | | 33.5 ± 0.8 **^a^** | 168.0 ± 4.4 **^b^** | 188.2 ± 4.3 **^b^** | 339.0 ± 6.4 **^b^** | | 191.3 ± 2.8 **^a^** |
|  |  | |  |  |  |  | |  |
| ***F66*** |  | |  |  |  |  | |  |
| 4 week | (n = 11) | | 15.0 ± 0.4 | 48.6 ± 1.7 **^b^** | 55.2 ± 1.4 **^b^** | 107.9 ± 3.3 **^b^** | | 75.3 ± 2.0 **^b^** |
| 10 week | (n = 7) | | 31.8 ± 0.5 **^b^** | 139.4 ± 3.0 **^b^** | 193.1 ± 9.6 **^b^** | 509.1 ± 17.8**^b^** | | 333.9 ± 9.5 **^b^** |
| 4 month | (n = 11) | | 35.7 ± 0.8 **^b^** | 149.9 ± 4.4 **^b^** | 199.9 ± 6.2 **^b^** | 507.7 ± 20.1**^b^** | | 347.9 ± 6.9 **^b^** |
| 6 month | (n = 9) | | 37.9 ± 0.8 | 164.9 ± 4.6 **^b^** | 220.3 ± 6.3 **^b^** | 552.3 ± 22.9**^b^** | | 345.7 ±11.3**^b^** |
| 8 month | (n = 9) | | 38.3 ± 1.0 | 162.7 ± 4.7 **^b^** | 219.2 ± 4.5 **^b^** | 563.7 ± 16.2**^b^** | | 359.0 ± 7.9 **^b^** |
|  |  | |  |  |  |  | |  |
| ***F66; mdx*** | | |  |  |  |  | |  |
| 4 week | (n = 9) | | 14.3 ± 0.4 **^c^** | 45.3 ± 0.9 **^e^** | 53.1 ± 1.5 **^d^** | 106.3 ± 3.0 **^e^** | | 76.4 ± 2.2 **^e^** |
| 10 week | (n = 11) | | 29.0 ± 0.5**^e,g^** | 146.5 ± 3.9 **^e^** | 162.4 ± 3.3**^e,f^** | 394.4 ± 8.1 **^e,h^** | | 270.0 ± 6.5 **^e,h^** |
| 4 month | (n = 9) | | 34.4 ± 1.3 **^d^** | 184.8 ± 9.3**^e,g^** | 212.0 ± 10.9**^d^** | 494.5 ± 24.5**^e^** | | 275.6 ±12.2**^e,h^** |
| 6 month | (n = 11) | | 38.1 ± 0.5 **^e^** | 213.6 ± 4.7**^e,h^** | 237.6 ± 4.9**^e,f^** | 570.5 ± 17.5**^e^** | | 298.0 ± 5.8 **^e,g^** |
| 8 month | (n = 9) | | 40.4 ± 1.2 **^e^** | 239.4 ± 6.7**^e,h^** | 237.3 ± 6.2**^e,f^** | 528.8 ± 15.3**^e^** | | 267.3 ±15.3**^d,h^** |
|  |  | |  |  |  |  | |  |

Comparison between age-matched groups. **^a^** *p <* 0.01 vs. *wt*; **^b^** *p <* 0.001 vs. *wt*; **^c^** *p* < 0.05 vs. *mdx*; **^d^** *p* < 0.01 vs. *mdx*; **^e^** *p* < 0.001 vs. *mdx*; **^f^** *p <* 0.05 vs. *F66*; **^g^** *p <* 0.01 vs. *F66*; **^h^** *p <* 0.001 vs. *F66*. Mean ± SEM.

**Table S3.** Serum creatine kinase (U/L) levels in *wt, F66*, *Dysf^-/-^,* and *F66; Dysf^-/-^* mice at the ages from 4 weeks to 8 months.

|  | 4 weeks | 10 weeks | 4 months | 6 months | 8 months |
| --- | --- | --- | --- | --- | --- |
|  |  |  |  |  |  |
| *wt* | 303 ± 99 (5) | 233 ± 49 (13) | 448 ± 59 (7) | 415 ± 85 (6) | 276 ± 47 (6) |
| *F66* | 267 ± 50 (8) | 277 ± 42 (10) | 546 ± 126 (5) | 440 ± 64 (6) | 285 ± 61 (5) |
| *Dysf ^-/-^* | 198 ± 21 (4) | 872 ± 89**^c^**  (11) | 1688 ± 318**^a^** (4) | 2187 ± 160**^c^** (4) | 1572 ± 86**^c^**  (14) |
| *F66; Dysf ^-/-^* | 540 ± 66**^d^** (9) | 3832 ± 397**^c,e^** (13) | 2914 ± 583**^b^** (6) | 1876 ± 240**^c^** (5) | 1826 ± 141**^c^**(13) |
|  |  |  |  |  |  |

**^a^** *p <* 0.05 vs. *wt*; **^b^** *p <* 0.01 vs. *wt*; **^c^** *p <* 0.001 vs. *wt*; **^d^** *p <* 0.01 vs. *Dysf ^-/-^*; **^e^** *p <* 0.001 vs. *Dysf ^-/-^*. Mean ± SEM.

**Table S4.** Serum creatine kinase (U/L) levels in *wt, F66,* *mdx,* and *F66; mdx* mice at the ages from 4 weeks to 8 months.

|  | 4 weeks | 10 weeks | 4 months | 6 months | 8 months |
| --- | --- | --- | --- | --- | --- |
|  |  |  |  |  |  |
| *wt* | 303 ± 99 (5) | 233 ± 49 (13) | 448 ± 59 (7) | 415 ± 85 (6) | 276 ± 47 (6) |
| *F66* | 267 ± 50 (8) | 277 ± 42 (10) | 546 ± 126 (5) | 440 ± 64 (6) | 285 ± 61 (5) |
| *mdx* | 1247 ± 198**^b^**(9) | 4395 ±1121**^b^** (10) | 4411±1168**^a^**(11) | 4275 ± 826**^b^**(10) | 7398±3202 (6) |
| *F66; mdx* | 2687 ± 603**^b^**(7) | 8198 ±1378**^c,d^**(10) | 3877 ± 773**^b^** (8) | 6646 ±2652**^a^**(10) | 2874 ± 522**^b^**(7) |
|  |  |  |  |  |  |

Comparison between age-matched groups. **^a^** *p <* 0.05 vs. *wt*; **^b^** *p <* 0.01 vs. *wt*; **^c^** *p <* 0.001 vs. *wt*; **^d^** *p <* 0.05 vs. *mdx*. Mean ± SEM. The sample size is shown in parenthesis.

**Table S5.** Evans blue dye (EBD) uptake in *wt, F66,* *Dysf^-/-^,* and *F66;Dysf^-/-^* mice at the age of 10 weeks.

|  |  | EBD absorbance/g (muscle weight) | | | | |
| --- | --- | --- | --- | --- | --- | --- |
|  |  | abdominal | quadriceps | gastrocnemius /soleus | gluteus/ hamstring | triceps |
|  |  |  |  |  |  |  |
| *wt* | (n = 8) | 1.81 ± 0.31 | 0.76 ± 0.10 | 0.64 ± 0.08 | 1.09 ± 0.08 | 0.80 ± 0.12 |
| *F66* | (n = 7) | 1.82 ± 0.22 | 1.11 ± 0.09**^a^** | 1.23 ± 0.09**^c^** | 1.18 ± 0.11 | 0.94 ± 0.06 |
| *Dysf ^-/-^* | (n = 6) | 2.77 ± 0.30**^a^** | 1.26 ± 0.07**^b^** | 1.00 ± 0.07**^b^** | 1.76 ± 0.26**^a^** | 1.33 ± 0.18**^a^** |
| *F66;Dysf ^-/-^* | (n = 7) | 3.37 ± 0.32**^b,d^** | 3.04 ± 0.28**^c,e,g^** | 1.57 ± 0.07**^c,d,g^** | 2.76 ± 0.19**^c,e,f^** | 2.52 ± 0.10**^c,e,g^** |
|  |  |  |  |  |  |  |

**^a^** *p <* 0.05 vs. *wt*; **^b^** *p <* 0.01 vs. *wt*; **^c^** *p <* 0.001 vs. *wt*; **^d^** *p <* 0.01 vs. *F66*; **^e^** *p <* 0.001 vs. *F66*; **^f^** *p* < 0.01 vs. *Dysf ^-/-^*; **^g^** *p* < 0.001 vs. *Dysf ^-/-^*. Mean ± SEM.

**Table S6.** Effect of ACVR2B/Fc on muscle weights (mg) in *wt* and *Dysf^-/-^* mice at the ages of 10 weeks, 8 months, and 14 months.

|  |  | **muscle weights (mg)** | | | |
| --- | --- | --- | --- | --- | --- |
|  |  | **pectoralis** | **triceps** | **quadriceps** | **gastrocnemius** |
| **10 week old** |  |  |  |  |  |
| ***wt*** |  |  |  |  |  |
| PBS | (n = 9) | 75.6 ± 2.3 | 93.0 ± 3.0 | 188.8 ± 6.2 | 131.4 ± 4.6 |
| ACVR2B/Fc | (n = 12) | 107.5 ± 3.5 **^a^** | 127.3 ± 3.7 **^a^** | 248.1 ± 7.3 **^a^** | 169.8 ± 3.7 **^a^** |
| Change, % |  | +42.2 | +36.9 | +31.4 | +29.2 |
| ***Dysf ^-/-^*** |  |  |  |  |  |
| PBS | (n = 6) | 80.2 ± 2.8 | 98.5 ± 2.7 | 213.0 ± 5.0 | 138.5 ± 3.7 |
| ACVR2B/Fc | (n = 10) | 118.0 ± 2.7 **^d^** | 131.1 ± 3.2 **^d^** | 278.9 ± 6.1 **^d^** | 180.5 ± 3.6 **^d^** |
| Change, % |  | +47.1 | +33.1 | +30.9 | +30.4 |
|  |  |  |  |  |  |
| **8 month old** |  |  |  |  |  |
| ***wt*** |  |  |  |  |  |
| PBS | (n = 10) | 98.8 ± 2.1 | 120.9 ± 2.2 | 247.6 ± 5.5 | 170.6 ± 3.7 |
| ACVR2B/Fc | (n = 6) | 137.4 ± 2.8 **^a^** | 157.9 ± 2.6 **^a^** | 310.1 ± 6.0 **^a^** | 203.0 ± 3.4 **^a^** |
| Change, % |  | +39.2 | +30.7 | +25.2 | +19.0 |
| ***Dysf ^-/-^*** |  |  |  |  |  |
| PBS | (n = 12) | 122.5 ± 4.8 | 134.3 ± 3.0 | 251.8 ± 5.0 | 161.6 ± 3.7 |
| ACVR2B/Fc | (n = 9) | 146.7 ± 2.5 **^c^** | 154.2 ± 3.6 **^d^** | 312.0 ± 12.6 **^d^** | 191.8 ± 4.4 **^d^** |
| Change, % |  | +20.0 | +14.8 | +23.9 | +18.7 |
|  |  |  |  |  |  |
| **14 month old** |  |  |  |  |  |
| ***Dysf ^-/-^*** |  |  |  |  |  |
| PBS | (n = 7) | 98.9 ± 3.4 | 115.4 ± 7.8 | 175.9 ± 9.1 | 147.9 ± 4.8 |
| ACVR2B/Fc | (n = 8) | 126.1 ± 9.5 **^b^** | 151.9 ± 9.8 **^b^** | 226.9 ± 20.0 **^b^** | 184.6 ± 7.0 **^c^** |
| Change, % |  | +27.5 | +31.6 | +29.0 | +24.9 |
|  |  |  |  |  |  |

Comparison between age-matched groups. **^a^** *p <* 0.001 vs. *wt* with PBS; **^b^** *p* < 0.05 vs. *Dysf ^-/-^* with PBS; **^c^** *p* < 0.01 vs. *Dysf ^-/-^* with PBS; **^d^** *p* < 0.001 vs. *Dysf ^-/-^* with PBS. Mean ± SEM.

**Table S7.** Serum creatine kinase (U/L) levels in ACVR2B/Fc-injected *wt* and *Dysf^-/-^* mice at the ages from 10 weeks to 14 months.

|  | 10 weeks | 8 months | 14 months |
| --- | --- | --- | --- |
|  |  |  |  |
| ***wt*** |  |  |  |
| PBS | 233 ± 49 (13) | 276 ± 47 (6) | - |
| ACVR2B/Fc | 143 ± 21 (11) | 295 ± 95 (5) | - |
|  |  |  |  |
| ***Dysf ^-/-^*** |  |  |  |
| PBS | 872 ± 89**^a^**  (11) | 1572 ± 86**^a^**  (14) | 1241 ± 92 (7) |
| ACVR2B/Fc | 1321 ± 96**^a,b^** (12) | 2338 ± 200**^a,b^**(10) | 1687 ± 101**^b^** (7) |
|  |  |  |  |

**^a^** *p <* 0.001 vs. *wt*; **^b^** *p <* 0.01 vs. *Dysf ^-/-^*. Mean ± SEM.

**Table S8.** Relative expression levels (fold change compared to *wt*) in *Dysf^-/-^, F66*, *F66; Dysf^-/-^*, and ACVR2B/Fc-injected *wt* and *Dysf^-/-^* mice at the ages 10 weeks.

|  | Fold change compared to *wt* | | | | |
| --- | --- | --- | --- | --- | --- |
| Gene (mouse gene ID) | *Dysf^-/-^* | *F66* | *F66; Dysf^-/-^* | ACVR2B/Fc-injected | |
|  |  |  |  | *wt* | *Dysf^-/-^* |
|  |  |  |  |  |  |
| Dysferlin related |  |  |  |  |  |
| AHNAK nucleoprotein (*Ahnak*) | 1.02 | 1.31 | 1.33 | 0.99 | 1.06 |
| annexin A1 (*Anxa1*) | 1.18 | 2.71 | 5.30 | 0.95 | 1.36 |
| annexin A2 (*Anxa2*) | 1.05 | 1.40 | 2.64 | 0.90 | 1.26 |
| calcium channel, voltage-dependent, L type, alpha 1S subunit (*Cacna1s*) | 1.00 | 1.02 | 0.87 | 1.00 | 0.95 |
| calpain 3 (*Capn3*) | 0.97 | 0.67 | 0.38 | 0.89 | 0.82 |
| calsequestrin 1 (*Casq1*) | 1.01 | 1.00 | 0.98 | 1.03 | 1.01 |
| caveolin 3 (*Cav3*) | 0.99 | 1.02 | 0.98 | 0.94 | 1.01 |
| dynein, cytoplasmic 1 light intermediate chain 2 (*Dync1li2*) | 1.03 | 1.03 | 1.01 | 0.97 | 0.98 |
| dysferlin (*Dysf*) | 0.31 | 1.23 | 0.30 | 1.06 | 0.35 |
| EH-domain containing 1 (*Ehd1*) | 0.91 | 0.93 | 0.92 | 0.92 | 0.99 |
| EH-domain containing 2 (*Ehd2*) | 1.04 | 1.20 | 1.31 | 1.17 | 1.09 |
| fer-1-like 4 (*Fer1l4*) | 0.93 | 0.87 | 0.82 | 0.98 | 1.04 |
| fer-1-like 6 (*Fer1l6*) | 0.86 | 1.09 | 0.75 | 0.94 | 0.87 |
| histone deacetylase 6 (*Hdac6*) | 0.96 | 1.00 | 1.06 | 1.00 | 1.03 |
| myoferlin (*Myof*) | 1.29 | 1.75 | 4.42 | 1.03 | 1.42 |
| myomesin 2 (*Myom2*) | 0.97 | 1.20 | 0.89 | 1.07 | 0.98 |
| otoferlin (*Otof*) | 0.97 | 0.93 | 0.87 | 1.02 | 1.04 |
| parvin, beta (*Parvb*) | 1.01 | 1.37 | 1.13 | 1.04 | 1.09 |
| S100 calcium binding protein A10 (*S100a10*) | 1.05 | 1.65 | 1.41 | 0.80 | 1.07 |
| S100 calcium binding protein A4 (*S100a4*) | 1.13 | 2.57 | 1.98 | 1.04 | 1.43 |
| tripartite motif-containing 72 (*Trim72*) | 1.02 | 1.47 | 1.36 | 0.91 | 0.92 |
| tubulin, alpha 1A (*Tuba1a*) | 1.13 | 1.08 | 2.56 | 0.96 | 1.35 |
| tubulin, alpha 1B (*Tuba1b*) | 1.12 | 1.03 | 2.99 | 0.98 | 1.40 |
| tubulin, alpha-like 3 (*Tubal3*) | 0.52 | 0.54 | 0.72 | 0.70 | 1.09 |
| tubulin, beta 2A class IIA (*Tubb2a*) | 1.11 | 1.07 | 2.31 | 0.93 | 1.29 |
| tubulin, beta 2B class IIB (*Tubb2b*) | 1.16 | 1.05 | 2.14 | 0.89 | 1.22 |
| tubulin, beta 5 class I (*Tubb5*) | 1.11 | 1.15 | 2.52 | 0.95 | 1.28 |
| tubulin, beta 6 class V (*Tubb6*) | 1.42 | 1.67 | 3.69 | 1.09 | 1.71 |

**Table S8.** *Continued*

|  | Fold change compared to *wt* | | | | |
| --- | --- | --- | --- | --- | --- |
| Gene (mouse gene ID) | *Dysf^-/-^* | *F66* | *F66; Dysf^-/-^* | ACVR2B/Fc-injected | |
|  |  |  |  | *wt* | *Dysf^-/-^* |
|  |  |  |  |  |  |
| Tgfb related |  |  |  |  |  |
| activin A receptor, type 1 (*Acvr1*) | 1.09 | 0.83 | 1.07 | 1.04 | 1.09 |
| activin A receptor, type 1B (*Acvr1b*) | 0.84 | 0.98 | 0.67 | 1.01 | 0.86 |
| activin A receptor, type IC (*Acvr1c*) | 0.73 | 0.53 | 0.62 | 0.78 | 0.92 |
| activin receptor IIA (*Acvr2a*) | 0.98 | 0.92 | 0.99 | 0.95 | 0.84 |
| activin receptor IIB (*Acvr2b*) | 0.97 | 0.89 | 0.78 | 1.01 | 1.01 |
| activin A receptor, type II-like 1 (*Acvrl1*) | 1.02 | 0.99 | 1.11 | 0.96 | 0.96 |
| anti-Mullerian hormone (*Amh*) | 0.88 | 0.96 | 0.82 | 1.02 | 1.01 |
| anti-Mullerian hormone type 2 receptor (*Amhr2*) | 1.01 | 0.96 | 0.84 | 0.97 | 1.01 |
| artemin (*Artn*) | 0.87 | 0.85 | 0.86 | 1.01 | 0.97 |
| bone morphogenetic protein 1 (*Bmp1*) | 1.11 | 1.23 | 2.07 | 0.96 | 1.12 |
| bone morphogenetic protein 10 (*Bmp10*) | 0.93 | 1.02 | 0.78 | 0.87 | 1.06 |
| bone morphogenetic protein 15 (*Bmp15*) | 0.97 | 1.00 | 0.95 | 0.96 | 1.01 |
| bone morphogenetic protein 2 (*Bmp2*) | 1.03 | 1.15 | 0.99 | 1.02 | 1.03 |
| bone morphogenetic protein 3 (*Bmp3*) | 0.90 | 0.85 | 0.77 | 1.01 | 1.02 |
| bone morphogenetic protein 4 (*Bmp4*) | 0.90 | 2.04 | 1.01 | 0.94 | 0.86 |
| bone morphogenetic protein 5 (*Bmp5*) | 1.13 | 1.97 | 1.73 | 0.93 | 0.95 |
| bone morphogenetic protein 6 (*Bmp6*) | 0.79 | 0.88 | 0.50 | 1.00 | 0.76 |
| bone morphogenetic protein 7 (*Bmp7*) | 0.90 | 0.87 | 0.80 | 0.98 | 1.03 |
| bone morphogenetic protein 8a (*Bmp8a*) | 0.90 | 0.82 | 0.70 | 0.89 | 0.96 |
| bone morphogenetic protein 8b (*Bmp8b*) | 0.93 | 0.89 | 0.90 | 0.98 | 0.98 |
| bone morphogenetic protein receptor, type 1A (*Bmpr1a*) | 1.03 | 0.79 | 0.74 | 0.93 | 0.85 |
| bone morphogenetic protein receptor, type 1B (*Bmpr1b*) | 1.03 | 0.95 | 0.86 | 0.96 | 0.90 |
| bone morphogenetic protein receptor, type II (*Bmpr2*) | 1.04 | 1.32 | 0.92 | 1.02 | 1.04 |
| cripto, FRL-1, cryptic family 1 (*Cfc1*) | 0.95 | 0.98 | 0.83 | 1.05 | 1.03 |
| follistatin (*Fst*) | 0.99 | 1.33 | 1.48 | 0.76 | 0.91 |
| follistatin-like 1 (*Fstl1*) | 1.34 | 1.74 | 2.77 | 1.05 | 1.41 |
| follistatin-like 3 (*Fstl3*) | 1.06 | 1.09 | 0.92 | 1.28 | 1.13 |
| follistatin-like 4 (*Fstl4*) | 1.03 | 1.04 | 0.94 | 1.05 | 1.13 |

**Table S8.** *Continued*

|  | Fold change compared to *wt* | | | | |
| --- | --- | --- | --- | --- | --- |
| Gene (mouse gene ID) | *Dysf^-/-^* | *F66* | *F66; Dysf^-/-^* | ACVR2B/Fc-injected | |
|  |  |  |  | *wt* | *Dysf^-/-^* |
|  |  |  |  |  |  |
| follistatin-like 5 (*Fstl5*) | 0.98 | 1.00 | 0.89 | 1.02 | 1.00 |
| growth differentiation factor 1 (*Gdf1*) | 0.92 | 0.90 | 0.82 | 0.88 | 0.95 |
| growth differentiation factor 10 (*Gdf10*) | 0.94 | 0.96 | 0.84 | 1.02 | 0.95 |
| growth differentiation factor 11 (*Gdf11*) | 0.93 | 6.90 | 2.98 | 1.60 | 1.26 |
| growth differentiation factor 15 (*Gdf15*) | 0.70 | 0.78 | 1.04 | 0.95 | 1.05 |
| growth differentiation factor 2 (*Gdf2*) | 0.99 | 0.89 | 0.76 | 1.00 | 0.97 |
| growth differentiation factor 3 (*Gdf3*) | 0.91 | 0.94 | 1.23 | 1.15 | 1.20 |
| growth differentiation factor 5 (*Gdf5*) | 0.73 | 0.87 | 0.88 | 1.08 | 1.06 |
| growth differentiation factor 6 (*Gdf6*) | 0.94 | 0.87 | 0.96 | 1.01 | 1.06 |
| growth differentiation factor 7 (*Gdf7*) | 1.04 | 1.01 | 0.94 | 1.04 | 1.08 |
| growth differentiation factor 9 (*Gdf9*) | 1.10 | 1.10 | 0.97 | 1.19 | 1.16 |
| glial cell line derived neurotrophic factor (*Gdnf*) | 0.99 | 0.76 | 1.00 | 1.03 | 1.11 |
| HGF-regulated tyrosine kinase substrate (*Hgs*) | 1.01 | 1.21 | 1.22 | 0.99 | 1.01 |
| inhibin alpha (*Inha*) | 0.95 | 2.11 | 1.21 | 1.17 | 1.25 |
| inhibin beta-A (*Inhba*) | 1.11 | 2.23 | 1.29 | 1.03 | 1.02 |
| inhibin beta-B (*Inhbb*) | 1.00 | 1.32 | 1.14 | 1.04 | 1.09 |
| inhibin beta-C (*Inhbc*) | 1.01 | 1.01 | 0.92 | 1.10 | 1.20 |
| inhibin beta E (*Inhbe*) | 0.90 | 0.89 | 0.86 | 1.01 | 1.14 |
| left right determination factor 1 (*Lefty1*) | 1.00 | 1.08 | 0.97 | 1.06 | 1.12 |
| left-right determination factor 2 (*Lefty2*) | 0.98 | 0.83 | 0.91 | 1.20 | 1.42 |
| myostatin (*Mstn*) | 0.92 | 1.39 | 0.48 | 1.12 | 0.82 |
| nodal (*Nodal*) | 0.95 | 0.93 | 0.89 | 1.04 | 1.05 |
| neurturin (*Nrtn*) | 0.89 | 0.88 | 0.79 | 1.05 | 1.09 |
| persephin (*Pspn*) | 0.76 | 0.78 | 0.76 | 0.90 | 0.86 |
| SMAD family member 1 (*Smad1*) | 1.01 | 1.10 | 1.23 | 1.02 | 1.11 |
| SMAD family member 2 (*Smad2*) | 0.99 | 1.08 | 1.15 | 0.93 | 0.98 |
| SMAD family member 3 (*Smad3*) | 1.20 | 0.92 | 1.08 | 1.05 | 1.14 |
| SMAD family member 4 (*Smad4*) | 0.99 | 0.90 | 0.87 | 0.95 | 0.90 |
| SMAD family member 5 (*Smad5*) | 0.95 | 0.86 | 1.00 | 0.93 | 0.94 |
| SMAD family member 6 (*Smad6*) | 1.06 | 1.19 | 1.03 | 1.24 | 1.22 |
| SMAD family member 7 (*Smad7*) | 1.04 | 1.24 | 1.04 | 1.09 | 1.24 |

**Table S8.** *Continued*

|  | Fold change compared to *wt* | | | | |
| --- | --- | --- | --- | --- | --- |
| Gene (mouse gene ID) | *Dysf^-/-^* | *F66* | *F66; Dysf^-/-^* | ACVR2B/Fc-injected | |
|  |  |  |  | *wt* | *Dysf^-/-^* |
|  |  |  |  |  |  |
| SMAD family member 9 (*Smad9*) | 1.21 | 9.95 | 3.58 | 1.88 | 1.09 |
| SMAD specific E3 ubiquitin protein ligase 1 (*Smurf1*) | 1.00 | 1.07 | 1.12 | 1.02 | 1.02 |
| SMAD specific E3 ubiquitin protein ligase 2 (*Smurf2*) | 1.05 | 0.94 | 1.01 | 1.00 | 0.94 |
| teratocarcinoma-derived growth factor 1 (*Tdgf1*) | 1.14 | 0.97 | 0.85 | 0.89 | 1.09 |
| transforming growth factor, beta 1 (*Tgfb1*) | 1.00 | 1.05 | 1.84 | 1.09 | 1.27 |
| transforming growth factor, beta 2 (*Tgfb2*) | 1.12 | 1.17 | 1.02 | 1.04 | 1.08 |
| transforming growth factor, beta 3 (*Tgfb3*) | 0.97 | 0.86 | 1.28 | 0.89 | 1.01 |
| transforming growth factor, beta receptor I (*Tgfbr1*) | 1.09 | 1.36 | 1.73 | 0.96 | 1.01 |
| transforming growth factor, beta receptor II (*Tgfbr2*) | 0.98 | 1.09 | 1.40 | 1.08 | 1.10 |
| transforming growth factor, beta receptor III (*Tgfbr3*) | 1.03 | 1.28 | 1.04 | 1.02 | 0.97 |
| tolloid-like (*Tll1*) | 1.15 | 1.00 | 1.22 | 1.32 | 1.16 |
| tolloid-like 2 (*Tll2*) | 0.93 | 1.99 | 0.86 | 1.03 | 1.08 |
| WAP, FS, Ig, KU, and NTR-containing protein 1 (*Wfikkn1*) | 1.02 | 0.99 | 0.92 | 0.94 | 1.23 |
| WAP, follistatin/kazal, immunoglobulin, kunitz and netrin domain containing 2 (*Wfikkn2*) | 0.98 | 0.71 | 0.57 | 0.93 | 0.87 |
| zinc finger, FYVE domain containing 9 (*Zfyve9*) | 1.01 | 1.00 | 0.74 | 1.00 | 0.94 |

**Table S9.** Enriched top biological processes from gene ontology (GO) analysis using 763 genes that were significantly differentially expressed (fold change > 2.0 and *P* < 0.01).

| GO Biological Process | Retrieved genes | Annotated genes | *P*-value |
| --- | --- | --- | --- |
|  |  |  |  |
| total | 763 | 23344 | 1 |
|  |  |  |  |
| **Response to stimulus** | 353 | 6099 | 3.78E-34 |
| Response to stress | 205 | 2685 | 1.18E-32 |
| Defense response | 119 | 1030 | 3.77E-34 |
| Inflammatory response | 73 | 495 | 1.57E-27 |
| Response to oxidative stress | 21 | 309 | 1.36E-03 |
| Response to wounding | 73 | 568 | 8.46E-24 |
| Wound healing | 49 | 303 | 1.54E-20 |
| Plasma membrane repair | 3 | 8 | 1.72E-03 |
|  |  |  |  |
| **Signaling** | 257 | 5676 | 2.00E-09 |
| Apoptotic Signaling pathway | 19 | 252 | 6.69E-04 |
| Cell surface receptor signaling pathway | 152 | 3616 | 5.05E-04 |
| Cytokine mediated signaling pathway | 14 | 178 | 2.21E-03 |
| Enzyme-linked receptor protein signaling pathway | 49 | 757 | 4.82E-06 |
| Transmembrane receptor protein serine/threonine  kinase signaling pathway | 19 | 312 | 7.28E-03 |
| Transmembrane receptor protein tyrosine kinase  signaling pathway | 31 | 465 | 1.57E-04 |
| Integrin-mediated signaling pathway | 18 | 73 | 1.52E-11 |
| Lipopolysaccharide-mediated signaling pathway | 7 | 43 | 4.48E-04 |
| Signal transduction by phosphorylation | 48 | 593 | 8.74E-09 |
| MAPK cascade | 36 | 449 | 8.07E-07 |
| ERK-1 and ERK-2 cascade | 22 | 200 | 7.23E-07 |
| Stress-activated MAPK cascade | 13 | 176 | 5.32E-03 |
|  |  |  |  |
| **Developmental process** | 259 | 4827 | 3.08E-18 |
| Blood vessel development | 44 | 459 | 2.10E-10 |
| Muscle structure development | 21 | 352 | 6.22E-03 |
| Hemopoiesis | 72 | 776 | 1.52E-15 |
| Syncytium formation by plasma membrane fusion | 12 | 53 | 1.06E-07 |
|  |  |  |  |
| **Immune system process** | 185 | 1545 | 8.88E-57 |
| Immune response | 81 | 529 | 1.19E-31 |
| Lymphocyte activation | 65 | 565 | 9.79E-19 |
| Myeloid leukocyte activation | 35 | 151 | 3.20E-20 |
| Myeloid leukocyte migration | 30 | 117 | 7.43E-19 |
| Immune effector process | 53 | 365 | 6.22E-20 |

**Table 9.** *Continued*

| GO Biological Process | Retrieved genes | Annotated genes | *P*-value |
| --- | --- | --- | --- |
|  |  |  |  |
| total | 763 | 23344 | 1 |
|  |  |  |  |
| **Localization** | 240 | 4376 | 7.57E-18 |
| Transport | 204 | 3736 | 1.17E-14 |
| Vesicle-mediated transport | 69 | 1021 | 9.72E-09 |
| Vesicle fusion | 7 | 85 | 2.12E-02 |
| Endocytosis | 46 | 458 | 1.66E-11 |
| Phagocytosis | 24 | 116 | 3.98E-13 |
| Cytokine secretion | 21 | 109 | 4.90E-11 |
| Calcium ion transport into cytosol | 15 | 108 | 2.36E-06 |
| Lipid localization | 27 | 198 | 3.66E-10 |
| Lipid storage | 8 | 54 | 3.45E-04 |
| Lipid transport | 20 | 153 | 1.41E-07 |
|  |  |  |  |
| **Locomotion** | 86 | 1071 | 1.16E-14 |
| Cell motility | 35 | 297 | 6.04E-11 |
| Cell migration | 78 | 909 | 6.75E-15 |
|  |  |  |  |
| **Growth** | 53 | 882 | 1.63E-05 |
| Cell growth | 24 | 347 | 4.98E-04 |
| Developmental growth | 25 | 347 | 2.08E-04 |
| Developmental growth involved in morphogenesis | 12 | 131 | 1.24E-03 |
| Endochondral bone growth | 5 | 18 | 2.21E-04 |
| Skeletal muscle tissue growth | 3 | 7 | 1.10E-03 |
|  |  |  |  |
| **Cellular component organization or biogenesis** | 168 | 3139 | 3.11E-11 |
| Protein complex assembly | 36 | 581 | 2.05E-04 |
| Protein polymerization | 16 | 181 | 3.11E-04 |
| Actin filament polymerization | 8 | 116 | 3.66E-02 |
| Protein complex subunit organization | 97 | 1288 | 1.10E-14 |
| Protein oligomerization | 38 | 454 | 1.35E-07 |
| Actin filament organization | 10 | 113 | 3.96E-03 |
| Membrane organization | 35 | 398 | 1.29E-07 |
| Membrane fusion | 8 | 108 | 2.54E-02 |
| Membrane invagination | 8 | 36 | 1.69E-05 |
| Plasma membrane organization | 7 | 80 | 1.57E-02 |
| Mitochondrial fusion | 2 | 16 | 9.47E-02 |
